# Supplementary material for: Deciphering the complex interplay: Heterogeneous, threshold, and mediation effects of trade openness on CO2 emissions in Africa
Source: PLoS One. 2024 Oct 23;19(10):e0309736. doi: 10.1371/journal.pone.0309736 (PMC11498691; doi:10.1371/journal.pone.0309736)
Supplement: S1 Appendix — (DOCX) [file pone.0309736.s001.docx]

Appendix

Table 7: List of African countries used in the Analysis

| Lower Income | Lower Middle Income | Upper Middle Income | High Income |
| --- | --- | --- | --- |
| Burundi | Angola | Botswana | Seychelles |
| Burkina Faso | Benin | Gabon |  |
| Central African Republic | Côte d’Ivoire | Equatorial Guinea |  |
| Congo, Dem. Rep. | Cameroon | Libya |  |
| Ethiopia | Congo, Rep. | Mauritius |  |
| Gambia | Comoros | Namibia |  |
| Madagascar | Cabo Verde | South Africa |  |
| Mali | Algeria |  |  |
| Mozambique | Egypt |  |  |
| Niger | Ghana |  |  |
| Rwanda | Guinea |  |  |
| Sudan | Kenya |  |  |
| Sierra Leone | Lesotho |  |  |
| Sudan | Morocco |  |  |
| Togo | Mauritania |  |  |
| Uganda | Nigeria |  |  |
|  | Senegal |  |  |
|  | Eswatini |  |  |
|  | Tunisia |  |  |
|  | Tanzania |  |  |
|  | Zambia |  |  |
|  | Zimbabwe |  |  |
