# Supplementary material for: Deciphering the complex interplay: Heterogeneous, threshold, and mediation effects of trade openness on CO2 emissions in Africa
Source: PLoS One. 2024 Oct 23;19(10):e0309736. doi: 10.1371/journal.pone.0309736 (PMC11498691; doi:10.1371/journal.pone.0309736)
Supplement: Attachment — Submitted filename: Response to Reviewers.docx [file pone.0309736.s002.docx]

**Date: 22, April 2024**

**TO: PLOS ONE**

**Subject: Submission of a revised version of the manuscript PONE-D-24-07425 for evaluation**

**Title:** Does Trade openness asymmetrically affect CO2 Emissions in Africa? An Investigation of the Heterogeneous and Mediating Effects

**Manuscript No.: PONE-D-24-07425**

^*^[jinenus2014@gmail.com @gmail.com](mailto:jinenus2014@gmail.com @gmail.com)/ [getachewm@wollegauniversity.edu.et](mailto:Email%3Agetachewm@wollegauniversity.edu.et)

Dear Reviewers,

I appreciate the reviewers for dedicating their time to evaluating my manuscript, conducting a thorough reading, and providing valuable suggestions and comments that have significantly enhanced the paper's quality. In revising the manuscript, I have meticulously considered all the raised comments and suggestions. I have tried to explain the modifications made in response to each comment in a concise manner. Below, I provide my response to each comment

# 1. Regarding your concern about the Introduction section

In accordance with your insightful feedback regarding the need for a clear and concise introduction, we have revised the introduction section accordingly (please see pages 1 and 2)

Thank you for your valuable feedback, which contributed to enhancing our paper

# 2. Regarding your suggestion to improve the paper's organization

Taking into account your excellent feedback, we have revised the paper's structure accordingly.

# 3. Regarding your suggestions on Methodology and Data

Based on your good suggestions on methodology section, we revised it, made it more detail, and structured. Thank you for your comments.

# 4. Regarding your suggestions about the choice of variables and indicators used in the analysis

In line with your comments to clearly define and justify the choice of variables and indicators used in the analysis, we have included separate subsection as “Data source and Descriptions” (the revision can be seen from the revised Manuscript). We appreciate your comment that substantially improved our work.

# 5. **Regarding your concern** robustness of the results

Based on your constructive comments, we have provided information on the robustness of the results using Fixed Effect, and dividing the the whole sample in to different sub-samples based on their income status and run separate regression. We really appreciate your suggestions. (see the robustness section).

# 6. On your concerns regarding the presentation of results with appropriate tables, graphs, and statistical significance levels

Following your good comments, we have checked the appropriateness of the presentation of results, tables, graphs and statistical significances and corrected accordingly.

# 7. Regarding your suggestions about the need for detail discussions of implications relating with existing literature

We have carefully checked your suggestions and incorporated your comments including the most recent citations (see page 15 on the revised manuscript). We have also included the change on the original manuscript as a track change written in red color. Thank you, indeed for your constructive comments.

# 8. Concerning your suggestions about literature Review.

We have updated the most recent literature, including the articles you have recommended us.

Thank you so much for your good comment.

# 9. Regarding your concern about the alignment of the discussions section with objectives and hypothesis

Based on your helpful comments, we have confirmed that the discussion section is in line with the research objectives and hypotheses. We truly appreciate your constructive feedbacks.

# 10. Concerning citations and references

In line with your comments, we have checked and verified that all sources are properly cited and referenced. Thank you so much for your comments.

# 11. Regarding your suggestion about editorial issues

Following your comments, we have carefully read the work and corrected the grammatical and language errors. Thank you very much.

# 12. Regarding your concern about the Theoretical background

Incorporating your comments into the work, we have significantly improved and strengthened the theoretical background.
