# Supplementary material for: Deciphering the complex interplay: Heterogeneous, threshold, and mediation effects of trade openness on CO2 emissions in Africa
Source: PLoS One. 2024 Oct 23;19(10):e0309736. doi: 10.1371/journal.pone.0309736 (PMC11498691; doi:10.1371/journal.pone.0309736)
Supplement: Attachment — Submitted filename: Response to reviewers.docx [file pone.0309736.s003.docx]

**Date: 23 July 2024**

**TO: PLOS ONE**

**Subject: Submission of a revised version of the manuscript PONE-D-24-07425**R1 **for evaluation**

**Title:** Does Trade openness asymmetrically affect CO2 Emissions in Africa? An Investigation of the Heterogeneous and Mediating Effects

**Manuscript No.:** PONE-D-24-07425R1

^*^[jinenus2014@gmail.com @gmail.com](mailto:jinenus2014@gmail.com @gmail.com)/ [getachewm@wollegauniversity.edu.et](mailto:Email%3Agetachewm@wollegauniversity.edu.et)

Dear academic Editors and Reviewers,

Thank you for dedicating your time to evaluating my manuscript, thoroughly reading it, and offering valuable suggestions and comments that have greatly improved its quality. During the revision process, I carefully considered all your feedback. I have addressed each comment and suggestion, making modifications accordingly. Below, I provide my detailed responses to each comment.

Additions to the original manuscript are indicated by a green highlight (….) and deletions are indicated by track change.

Furthermore, I have revised the title of the manuscript to reflect the additions and improvements based on the feedback from the editors and reviewers and the updated content in the revised manuscript. The modified Title is:

“Deciphering the Complex Interplay: Heterogeneous, Threshold, and Mediation Effects of Trade Openness on CO2 Emissions in Africa”

# I. Response to academic editor’s Comments

# 1. Regarding your concern about the Motivation

Dear academic editor/s!

We really thank you for your comments on the need of clearly articulating the motivation of the study. The motivation behind the study is articulated in the introduction section as follows. Thank you, indeed!

- Africans are already disproportionately experiencing the negative consequences of climate change more severely than other continents, despite bearing the least responsibility for the problem.
- Existing studies focus on homogeneous and linear relationship between the two variables.
- The motivation behind this study is therefore; to investigate the heterogeneous, nonlinear effect of Trade openness on CO2 emissions and the mediation mechanisms through which trade openness affects CO2 emissions in Africa. These approaches are overlooked in African studies (These justifications are included in the introduction section of our manuscript)

Thank you for your valuable feedback, which contributed to enhancing our paper

# 2. Regarding your suggestion to improve the Contribution

Dear editor/s, thank you so much for your comment that improved our paper. Based on your good comments, we have included the contribution of our paper to our revised manuscript clearly in detail (see end of the introduction section of our manuscript).

Thank you indeed!

# 3. Concerning your suggestions on novelty of methodology and data

Regarding your suggestion on the need of highlighting the novelty in the data and Methodology, following your suggestions and good comments, we have discussed that the study undertaken a comprehensive analysis using Quantile regression, threshold approaches and mediation analysis employing a recent data (please see the introduction and methodology section of our revised manuscript).

# 4. Regarding your concern on the Theoretical Frameworks

In line with your comment, we have revised the theoretical framework and included updated theories. Please refer to green highlighted under the “Theoretical framework” section. Thank you for your valuable feedback.

# 5. On your concerns regarding the methodology

Following your good comments to include non-linear relationship between trade openness and CO2 emissions, this study employs the panel threshold approach. The incorporated panel threshold model is indicated by equation (6) under methodology section and the threshold regression result is incorporated under the “Threshold Regression” of the revised manuscript. Thank you indeed!

# II. Response to reviewer comments

# 1. Regarding your concern about the methodology used in robustness check

We have removed this section and replaced by the “panel threshold effect model” to investigate the nonlinear effects of trade openness on CO2 emissions.

# 2. Concerning your suggestions about test of cross-sectional dependence.

Thank you so much for your good comment. Concerning your suggestions about testing for cross-sectional dependence, we have addressed this in the revised manuscript. We conducted tests for cross-sectional dependence and included the results in the analysis section

# 3. Regarding your suggestion about co-integration test for long run relationships

Dear reviewer/s! We did not conduct cointegration test for long run relationship since the variables are found to be stationary at level. We truly appreciate your constructive feedbacks.

# 4. Concerning suggestions about the need for strengthening the comparisons of our findings with with the literature.

Regarding the suggestions to enhance the comparisons of our findings with the existing literature, we have made the necessary adjustments. Thank you for your valuable feedback

# 5. Regarding your suggestion about the need to include limitations and areas of future research

Dear reviewer!

Following your good comments, on the need of commenting on the extent to which the approach taken in the research enables certain generalizations to be made, gaps revealed and areas of future research, we have included a separate subsection as “Limitations and areas of further research”.

Thank you for providing us such suggestions that has enhanced the quality of manuscript.
